# Empirical Nonnegative Finite-Resolution MAR-PID for Continuous Variables

**DOI:** 10.3390/e28060641

**Published:** 2026-06-06

**Authors:** András Telcs

**Affiliations:** HUN-REN Wigner Research Centre for Physics, Konkoly Thege Miklós út 29-33, 1121 Budapest, Hungary; telcs.szit.bme@gmail.com

**Keywords:** partial information decomposition, MAR-PID, Blackwell order, recursive quantile binarization, continuous variables, zonogons, empirical estimation

## Abstract

We develop a finite-resolution empirical framework for applying nonnegative Mages–Anastasiadi–Rohner partial information decomposition (MAR-PID) to continuous and non-binary discrete variables. The variables are represented by recursive quantile binarization. This provides a balanced binary-tree representation at each finite depth. MAR-PID is then applied to binary target components, and the resulting atoms are aggregated back to the original target and source variables. The construction gives nonnegative target-relative information summaries for observed variables up to the chosen empirical resolution. The pipeline consists of conditional channel estimation, bit-level MAR-PID computation, projection of source atoms to the original variables, and aggregation of the resulting information contributions. The obtained quantities are empirical estimates of finite-resolution population quantities. XOR and mixed redundancy–synergy examples show how the representation separates informational mechanisms, which signed interaction-information summaries can conflate. We focus here on the finite-resolution MAR-PID construction and its information-level quantities. Downstream summaries, such as resolution-normalized PID-dimension descriptors and thresholded support-based degrees of freedom, are indicated but left for separate work.

## 1. Introduction

Understanding information flow in multivariate systems is a central problem in the analysis of complex data. In applications such as neuroscience, climate science, economics, and networked dynamical systems, observed signals arise from several interacting components. Pairwise measures are often insufficient in such settings, because information about a target may be redundant across sources, unique to one source, or available only through joint observation.

Classical multivariate information measures, such as interaction information, are useful algebraic summaries but can take negative values. This is not an error: the sign reflects the alternating-sum structure of the definition and the fact that redundancy and synergy contribute with opposite signs. Nevertheless, signed net quantities can be difficult to interpret when the aim is to identify distinct informational mechanisms.

Partial information decomposition (PID) addresses this issue by decomposing information about a chosen target into redundant, unique, and synergistic components. Early PID approaches introduced important conceptual distinctions but did not fully resolve the problem of obtaining nonnegative atoms in general multivariate settings. Mages, Anastasiadi, and Rohner [1] (see recent references in that paper) introduced a full-lattice PID based on the Blackwell order and the geometry of binary-input channels. We refer to this construction as MAR-PID. Its central advantage is that the resulting partial information atoms are nonnegative for a broad class of information functionals.

Partial information decomposition was introduced to separate redundant, unique, and synergistic contributions to the information that several sources provide about a target [2,3]. Since then, a number of alternative approaches to redundancy, synergy, and multivariate dependence have been developed, including optimization-based, geometric, pointwise, and dual-decomposition formulations [4]. Related work has also emphasized the role of synergy in information modification and dynamical (see also references there) systems, as well as the limitations of classical Shannon-type summaries for describing higher-order dependence [5] (see also references in the cited paper). The present paper does not aim to compare these PID measures. We use the Blackwell-order construction of Mages, Anastasiadi, and Rohner because it provides nonnegative partial information atoms for a broad class of information functionals and is naturally formulated in terms of binary-input channel geometry [1,6]. The binary-input channel geometry, Blackwell order, zonogon representation, cumulative loss construction, and nonnegativity theorem are inherited from MAR-PID. The new contribution of the present paper is the finite-resolution empirical layer needed when the variables of interest are continuous or high-cardinality rather than already given in a finite binary form. The comparison made here is conceptual rather than benchmark-based: different PID proposals define different decompositions, and a numerical comparison would require separate choices of estimators, test distributions, and error criteria.

The basic idea is to represent variables by binary coordinates obtained from recursive quantile, or median, partitioning. For a continuous target variable, the finite-resolution representation is a vector of binary target components. MAR-PID is applied to each binary target component, and the resulting atomic information quantities are aggregated over target bits. For sources that are also represented by binary coordinates, the bit-level atoms are pushed forward to atoms indexed by the original source variables. Thus the binary representation acts as an intermediate computational device, while the final summaries are reported at the level of the original variables.

The resulting object is a finite-resolution MAR-PID summary induced by the chosen binary representation, not a representation-independent PID of the underlying continuous variable. This distinction is important: the paper extends the empirical use of MAR-PID to finite-resolution representations of continuous or high-cardinality observations, but it does not modify the MAR-PID nonnegativity theorem itself.

This construction should also be distinguished from set-based structural degrees of freedom. If a discretized dynamical system is known explicitly, and if one knows the set of present-time variables influencing each next-time component, then set cardinalities and set-theoretic inclusion-exclusion methods provide direct structural descriptors. In observational time-series analysis, however, the update rule and its influence sets are usually not available. The present paper does not attempt to define degrees of freedom from such observations. Instead, it provides empirical nonnegative MAR-PID atoms that can later serve as the basis for support-level or scale-based summaries.

### Contributions

The main contributions of this paper are as follows:

- A finite-resolution binary representation of continuous or multilevel variables based on recursive quantile binarization;
- A conditional dyadic property showing that, at every fixed finite-tree level, the recursive quantile code produces balanced and conditionally independent binary coordinates under non-atomic conditional laws;
- A bit-level empirical procedure that applies MAR-PID to binary target components obtained from the finite-resolution representation;
- An aggregation scheme over resolved target bits, producing finite-resolution target-bit summaries for the original target variable;
- A source-side pushforward that maps bit-level MAR-PID atoms to summaries indexed by atoms over the original source variables;
- Illustrative examples showing how nonnegative MAR-PID atoms represent XOR-type synergy and mixed redundancy–synergy mechanisms.

Although this paper focuses on the empirical construction and aggregation of MAR-PID atoms, the procedure is not tied to one side of the redundancy–synergy distinction. The finite-resolution binarization, target-bit aggregation, and source-side pushforward apply to the resulting atoms whether they are interpreted as redundant, unique, or synergistic contributions.

The present paper deliberately remains at the information decomposition level. PID-based dimensions and support-based degrees-of-freedom summaries require additional choices concerning scale normalization, activity thresholds, and source/target-bit aggregation. These questions are natural continuations of the present construction and are left to a separate treatment.

The structure of the paper reflects this separation. Section 2 recalls only the MAR-PID background needed for the construction. Section 3 defines the finite-resolution binary representation and proves the conditional dyadic property. Section 4 defines the bit-level MAR-PID aggregation and the pushforward to original-variable atoms. Section 5 presents the empirical plug-in computation. Section 6 presents illustrative examples, and Section 7 discusses interpretation, limitations, and subsequent support- or scale-based summaries.

## 2. MAR-PID Background

We briefly recall the objects needed for the empirical construction. Let

$$V=\{V_{1},\ldots ,V_{n}\}$$

be a finite collection of observed variables, and let *T* denote the target. A source is a nonempty subset S⊆V. We write

S=P(V)∖{∅}

for the set of all sources. An atom is an antichain in S; that is, a collection α⊆S such that no source in α is strictly contained in another. The set of atoms is denoted by A(V).

The MAR-PID construction assigns to each atom α∈A(V) a partial information contribution about the target. These atoms are obtained by applying Möbius inversion to a cumulative loss functional on the synergy/loss lattice. We write

$$I_{\cup ,f}\left(\alpha ;T\right)$$

for the cumulative loss and

$$\Delta I_{\cup ,f}\left(\alpha ;T\right)$$

for its Möbius inverse. When the functional *f* is fixed, we suppress *f* when no confusion is possible.

The construction is based on comparing channels in the Blackwell order. For a source *S* and a binary target T∈{0,1}, the relevant channel is

$$\kappa_{S}\left(s|t\right)=P\left(S=s|T=t\right),\;t\in \left\{0,1\right\}.$$

For binary-input channels, the Blackwell order admits a zonogon representation, which makes joins and meets computable and supplies the geometric inequality behind the nonnegativity of the atoms. The formal lattice and channel definitions are recalled in Appendix A and Appendix B.

We use the synergy/loss orientation of the MAR-PID lattice throughout. Thus ${⪯}_{S}$ denotes the order on antichains used for cumulative loss and Möbius inversion, as recalled in Appendix A. The symbol ∨ in $\kappa^{\vee }\left(\alpha \right)$ denotes the Blackwell join of the source channels $\left\{\kappa_{S}:S\in \alpha \right\}$. For binary-input channels this join is represented and computed through the associated zonogon geometry; the zonogon construction is therefore a geometric representation of the Blackwell join, not a separate source lattice operation.

The functional $i_{f}\left(p,\kappa \right)$ denotes the information functional used in MAR-PID for a binary-input channel κ and target bias p=P(T=1). The cumulative quantity used below is a loss relative to the full source channel:

$$I_{\cup ,f}\left(\alpha ;T\right)=i_{f}\left(p,\kappa_{V}\right)-i_{f}\left(p,\kappa^{\vee }\left(\alpha \right)\right).$$

Partial information atoms are then obtained by Möbius inversion with respect to ${⪯}_{S}$. We assume *f* belongs to the admissible class covered by the MAR-PID nonnegativity theorem; the present paper does not modify that theorem or enlarge its scope.

A useful way to view the MAR-PID result is that it provides nonnegative target-relative atoms. In the binary-target case, these atoms satisfy

$$\Delta I_{\cup ,f}\left(\alpha ;T\right)\geq 0,\;\alpha \in A\left(V\right).$$

For finite multilevel targets, MAR-PID can be formulated through pointwise binary-input decompositions over target states. In the empirical construction developed below, we use binary components of a finite-resolution representation of the target. This keeps every computational step inside the binary-input setting while allowing the final summaries to refer to the original target variable after aggregation.

## 3. Recursive Quantile Binarization

In practice, variables may be continuous or have many possible values. We therefore introduce a finite-resolution binary representation. The construction is based on recursive quantile (median) partitioning. For simplicity of notation, assume first that a variable *X* takes values in [0,1]; other one-dimensional variables may be transformed to this case by a monotone distributional transform or by empirical ranks.

Let *x* be a realization of *X*.

1. Set $A^{\left(0\right)}=\left[0,1\right]$.
2. For k=1,2,…:
  (a) Choose the median $m^{\left(k-1\right)}$ of *X* conditional on $X\in A^{\left(k-1\right)}$;
  (b) Set
    $$B_{k}\left(x\right)=\left\{\begin{array}{cc} 0, & x\leq m^{\left(k-1\right)},\\ 1, & x>m^{\left(k-1\right)}; \end{array}\right.$$
  (c) Update
    $$A^{\left(k\right)}=\left\{\begin{array}{cc} A^{\left(k-1\right)}\cap \left(-\infty ,m^{\left(k-1\right)}\right], & B_{k}\left(x\right)=0,\\ A^{\left(k-1\right)}\cap \left(m^{\left(k-1\right)},\infty \right), & B_{k}\left(x\right)=1. \end{array}\right.$$
3. Output the binary sequence ${\left(B_{k}\left(x\right)\right)}_{k\geq 1}$.

In empirical work, the medians are estimated from samples. For time-series prediction or conditional analysis, the medians may be conditional on past information or on a conditioning state. The following proposition records the finite-tree-level property used in the construction.

**Proposition 1** 
(Conditional dyadic structure)**.** *Fix n≥0 and write $F_{n}=\sigma \left(X_{n}\right)$. Assume that the recursive conditional median binarization of $X_{n+1}$ relative to $F_{n}$ is well defined up to every finite depth. Then, for every K≥1 and every binary word $\left(b_{1},\ldots ,b_{K}\right)\in {\left\{0,1\right\}}^{K}$,*

$$P\left(B_{1}^{n+1}=b_{1},\ldots ,B_{K}^{n+1}=b_{K}|F_{n}\right)=2^{-K}\;a.s.$$

*Consequently, for every fixed K, the variables $B_{1}^{n+1},\ldots ,B_{K}^{n+1}$ are conditionally independent given $F_{n}$, and each is conditionally Bernoulli(1/2).*

The proof is given in Appendix C. This statement can be viewed as a finite-depth dyadic analogue of the Rosenblatt transform [7]. The construction produces balanced binary components, which is useful both statistically and computationally.

Proposition 1 is used here as a finite-resolution coding result. Recursive conditional median splitting produces balanced binary coordinates with a dyadic finite-tree structure. This gives each resolved target coordinate a common binary information scale and makes it suitable as a binary target for MAR-PID. The target-bit aggregation and the projection to original-variable atoms are separate construction steps introduced below.

**Remark 1** 
(Representation dependence)**.** *The construction is multivariate at the PID level: MAR-PID is applied to a collection of source variables and a target. The recursive quantile binarization step, however, is specified here for scalar variables. A vector-valued observed variable can be included either by treating its coordinates as separate observed variables, or by first choosing an additional finite-resolution encoding of the vector. Different such encodings may lead to different finite-resolution MAR-PID summaries.*

*For scalar variables, the quantile construction is invariant under strictly monotone transformations at the population level, or under rank-based empirical implementation, because the induced order of observations is unchanged. The finite-depth dyadic property in Proposition 1 requires the conditional median splits used in the construction; unconditional quantiles would not in general yield the same conditional dyadic property. Ties or atoms require a deterministic or randomized tie-breaking convention, and the exact balanced-split statement should be understood under the stated non-atomic conditional-law assumption.*

The use of recursive median splits is part of the finite-resolution representation. Median splitting is the choice used in Proposition 1, since it gives balanced binary coordinates and the finite-depth dyadic property. Other quantile splits may be useful for application-specific encodings—for example, to give more resolution to tail events—but they define different finite-resolution summaries and need not satisfy the same dyadic property. For scalar variables, the rank-based median construction is invariant under strictly monotone transformations, apart from tie-handling conventions. For vector-valued variables, or for non-median encodings, the chosen representation should be reported.

## 4. Bit-Level MAR-PID and Lifting to Original Variables

The binary variables introduced above are computational intermediates. We now describe how MAR-PID is applied at the bit level and how the resulting atoms are aggregated back to finite-resolution summaries for the original variables.

Let *Y* be an original target variable, and let

$$Y^{\left(K\right)}=\left(C_{1},\ldots ,C_{K}\right)$$

be its finite-resolution binary representation. Similarly, suppose that each original source variable $X_{i}$ is represented at a common source depth *k* by

$$X_{i}^{\left(k\right)}=\left(B_{i,1},\ldots ,B_{i,k}\right).$$

The binary source universe is

$$B=\{B_{i,r}:1\leq i\leq n,\;1\leq r\leq k\}.$$

For simplicity we use a common source depth *k* and target depth *K*. Allowing source-dependent depths $k_{i}$ is straightforward, but adds notation without changing the construction. For each binary target component $C_{\ell }$, MAR-PID is applied to the source universe B and target $C_{\ell }$. This gives bit-level atoms

$$\Delta I\left(\alpha ;C_{\ell }\right),\;\alpha \in A\left(B\right).$$

If Shannon information is used, the finite-resolution contribution associated with a bit-level atom α is aggregated over target bits by

$$\Delta I\left(\alpha ;Y^{\left(K\right)}\right):=\sum_{\ell =1}^{K}\Delta I\left(\alpha ;C_{\ell }\right).$$

The normalized version is

$$\bar{\Delta I}\left(\alpha ;Y^{\left(K\right)}\right):=\frac{1}{K}\sum_{\ell =1}^{K}\Delta I\left(\alpha ;C_{\ell }\right).$$

Throughout the paper, an overline denotes normalization by the number of resolved target bits.

When the sources are represented by binary coordinates, bit-level atoms can also be projected back to original source variables. Define the projection map on source-bit subsets by

$$\pi \left(S\right):=\{i:S\cap \left\{B_{i,1},\ldots ,B_{i,k}\right\}\neq \emptyset \},\;S\subseteq B.$$

For an atom α∈A(B), the collection {π(S):S∈α} may fail to be an antichain, because distinct bit-level sources can project to nested original-variable sources. We therefore define $\pi^{*}\left(\alpha \right)$ to be the antichain obtained by removing every projected set that is strictly contained in another projected set.

For an original-variable atom $\gamma \in A(\left\{X_{1},\ldots ,X_{n}\right\})$, define

$$\Delta I^{\mathrm{orig}}\left(\gamma ;C_{\ell }\right):=\sum_{\alpha :\pi^{*}\left(\alpha \right)=\gamma }\Delta I\left(\alpha ;C_{\ell }\right).$$

Aggregating over target bits gives

$$\Delta I^{\mathrm{orig}}\left(\gamma ;Y^{\left(K\right)}\right):=\sum_{\ell =1}^{K}\Delta I^{\mathrm{orig}}\left(\gamma ;C_{\ell }\right),$$

and the normalized version is

$${\bar{\Delta I}}^{\mathrm{orig}}\left(\gamma ;Y^{\left(K\right)}\right):=\frac{1}{K}\sum_{\ell =1}^{K}\Delta I^{\mathrm{orig}}\left(\gamma ;C_{\ell }\right).$$

Thus all bit-level atoms projecting to the same original-variable atom are summed. The resulting γ-indexed quantities are pushforward summaries of genuine bit-level MAR-PID atoms. This preserves, for each target component, the total MAR-PID information mass and keeps the projected quantities nonnegative. The projection is also a coarsening step: distinct bit-level mechanisms may project to the same original-variable atom and are then no longer separated at the original-variable level.

Aggregating over target bits gives a finite-resolution target-bit summary for the original target variable. The normalized version is an average information contribution per target bit, not a support or variable-count summary. In this sense, the binary coordinates are computational intermediates: MAR-PID is computed at the binary-coordinate level, while the final summaries are indexed by atoms over the original variables.

**Remark 2** 
(Scope of the target-bit aggregation)**.** *The source-side projection described above is a pushforward of nonnegative MAR-PID atoms. The subsequent summation over ℓ=1,…,K is a finite-resolution target-bit aggregation: it records how MAR-PID information about the resolved target coordinates is distributed over original-variable atoms. This target-bit summary is distinct from a native MAR-PID of the full target block $Y^{\left(K\right)}$. Cross-target-bit informational mechanisms require a separate target-block analysis or an additional support-level or dimension-level construction.*

## 5. Algorithmic Computation and Estimation

We now summarize the empirical computation. Throughout this section the target is a binary component $C_{\ell }\in \left\{0,1\right\}$ and the sources are finite-valued variables, either original discrete variables or binary coordinates obtained from the representation above.

### 5.1. Empirical Meaning and Statistical Scope

In this paper, the term empirical means that the construction starts from a finite observed sample and produces computable finite-resolution MAR-PID summaries after binarization and channel estimation. The resulting quantities are plug-in summaries of the chosen finite-resolution representation.

Their numerical values depend on the available sample, the binarization depths, and the set of source atoms included in the computation. Statistical error control, optimal resolution selection, and finite-sample confidence statements are separate questions and are not addressed here.

For reproducibility, the sample size *N*, target depth *K*, source depths $k_{i}$, tie-handling convention, and chosen source universe should be reported with any finite-sample computation.

### 5.2. Channel Estimation

Given samples

$${\left\{\left(c_{\ell }^{\left(m\right)},b^{\left(m\right)}\right)\right\}}_{m=1}^{N}$$

from the binary target $C_{\ell }$ and the source universe, estimate

$$p=P(C_{\ell }=1)$$

by

$$\hat{p}=\frac{1}{N}\sum_{m=1}^{N}1_{\left\{c_{\ell }^{\left(m\right)}=1\right\}}.$$

For each source *S* and each value $s\in A_{S}$, estimate

$$P\left(S=s|C_{\ell }=t\right),\;t\in \left\{0,1\right\}.$$

Let $N_{s,t}$ be the number of observations with S=s and $C_{\ell }=t$, and let $N_{t}$ be the number of observations with $C_{\ell }=t$. We use the plug-in estimator

$$\hat{P}\left(S=s|C_{\ell }=t\right)=\frac{N_{s,t}}{N_{t}}.$$

These conditional probabilities define the empirical channel ${\hat{\kappa }}_{S}$ for each source *S*. In the remainder of this section, all channels and MAR-PID quantities are empirical plug-in quantities unless explicitly stated otherwise; hats are omitted to lighten the notation.

Regularized channel estimators may be useful in sparse empirical applications, but their choice is an implementation issue and is not part of the finite-resolution MAR-PID construction studied here.

### 5.3. Zonogons, Joins, and Cumulative Loss

For each empirical channel ${\hat{\kappa }}_{S}$, construct the binary-input zonogon associated with its column vectors. Blackwell joins of channels are computed geometrically at the level of these zonogons. For an atom α, write

$$\kappa_{\vee }\left(\alpha \right)=\underset{S\in \alpha }{⋁}\kappa_{S}.$$

Let $\kappa_{V}$ denote the channel associated with the full source universe. The empirical cumulative loss is

$$I_{\cup ,f}\left(\alpha ;C_{\ell }\right)=i_{f}\left(p,\kappa_{V}\right)-i_{f}\left(p,\kappa_{\vee }\left(\alpha \right)\right).$$

Möbius inversion on the synergy/loss lattice gives

$${\Delta I}_{\cup ,f}\left(\alpha ;C_{\ell }\right)=I_{\cup ,f}\left(\alpha ;C_{\ell }\right)-\sum_{\beta {≺}_{S}\alpha }{\Delta I}_{\cup ,f}\left(\beta ;C_{\ell }\right).$$

The atoms are evaluated in an order compatible with ${⪯}_{S}$.

### 5.4. Aggregation over Target Bits and Projection to Original Variables

Repeat the binary-target computation for ℓ=1,…,K. The empirical block-level contribution is

$$\Delta I\left(\alpha ;Y^{\left(K\right)}\right)=\sum_{\ell =1}^{K}\Delta I\left(\alpha ;C_{\ell }\right),$$

or, in normalized form,

$$\bar{\Delta I}\left(\alpha ;Y^{\left(K\right)}\right)=\frac{1}{K}\sum_{\ell =1}^{K}\Delta I\left(\alpha ;C_{\ell }\right).$$

If source variables were represented by binary coordinates, one may additionally group bit-level atoms by their projected original-variable atom $\pi^{*}\left(\alpha \right)$ as in Section 4. This yields empirical original-variable summaries

$${\Delta I}^{\mathrm{orig}}\left(\gamma ;Y^{\left(K\right)}\right)=\sum_{\ell =1}^{K}\sum_{\alpha :\;\pi^{*}\left(\alpha \right)=\gamma }\Delta I\left(\alpha ;C_{\ell }\right).$$

### 5.5. Algorithmic Summary

The computation proceeds as follows.

1. Choose finite resolutions for source and target variables.
2. Represent the original target *Y* by binary components $C_{1},\ldots ,C_{K}$.
3. Represent continuous or multilevel source variables by binary coordinates when required.
4. For each target bit $C_{\ell }$, estimate the binary-input channels $\kappa_{S}$ for all sources *S* under consideration.
5. Construct the associated zonogons and compute Blackwell joins.
6. Compute empirical cumulative losses $I_{\cup ,f}\left(\alpha ;C_{\ell }\right)$.
7. Apply Möbius inversion to obtain ${\Delta I}_{\cup ,f}\left(\alpha ;C_{\ell }\right)$.
8. Aggregate over target bits to obtain $\Delta I(\alpha ;Y^{\left(K\right)})$ or its normalized version.
9. If source variables were binarized, project bit-level atoms to original-variable atoms and aggregate the corresponding contributions.

## 6. Illustrative Examples

The examples below are controlled mechanism checks rather than empirical benchmarks. They are chosen so that the redundant, unique, or synergistic structure is known in advance. This makes it possible to check whether the finite-resolution MAR-PID construction assigns nonnegative information contributions to the expected atoms. Real-data applications require further choices, including preprocessing, source selection, resolution choice, and statistical stabilization. These choices are application dependent and are not part of the construction studied here.

We provide three examples illustrating the finite-resolution MAR-PID viewpoint. The first contrasts the negative value of classical interaction information with the nonnegative PID representation of an XOR structure. The second shows that redundant and synergistic mechanisms may coexist in a binary-target distribution. The third illustrates the treatment of a non-binary discrete target.

### 6.1. XOR Under the Doubling Map

Consider the doubling map on [0,1),

M(u)=2umod1.

Write the binary expansion, ignoring the null set of dyadic rationals, as

$$u=0.u_{0}u_{1}u_{2}\ldots ,\;u_{i}\in \left\{0,1\right\}.$$

Then *M* acts as the left shift on binary digits.

Let $X_{0}$ and $Y_{0}$ be independent uniformly distributed points in [0,1), with binary expansions

$$X_{0}=0.b_{0}b_{1}b_{2}\ldots ,\;Y_{0}=0.c_{0}c_{1}c_{2}\ldots ,$$

where ${\left(b_{i}\right)}_{i\geq 0}$ and ${\left(c_{i}\right)}_{i\geq 0}$ are independent i.i.d. Bernoulli(1/2) sequences. Define

$$Z_{0}=0.\left(b_{0}⊕c_{0}\right)\left(b_{1}⊕c_{1}\right)\left(b_{2}⊕c_{2}\right)\ldots ,$$

where ⊕ denotes addition modulo two. Let

$$X_{t}=M^{t}\left(X_{0}\right),\;Y_{t}=M^{t}\left(Y_{0}\right),\;Z_{t}=M^{t}\left(Z_{0}\right).$$

Then $Z_{t}=X_{t}⊕Y_{t}$ digitwise for every t≥0.

At resolution $\delta =2^{-k}$, let $X_{t}^{\left(k\right)},Y_{t}^{\left(k\right)},Z_{t}^{\left(k\right)}$ denote the *k*-bit prefixes. Since

$$Z_{t}^{\left(k\right)}=X_{t}^{\left(k\right)}⊕Y_{t}^{\left(k\right)}$$

digitwise, the classical trivariate interaction information satisfies

$$I(X_{t}^{\left(k\right)};Y_{t}^{\left(k\right)};Z_{t}^{\left(k\right)})=-k$$

when information is measured in bits. Thus the signed interaction information summary is negative.

In the MAR-PID representation, keep the sources

$$V_{1}=X_{t}^{\left(k\right)},\;V_{2}=Y_{t}^{\left(k\right)},$$

and choose a binary target given by one output digit of $Z_{t}$.

In this example we take the source universe to be $V=\{V_{1},V_{2}\}$, where each $V_{i}$ is a *k*-bit finite-valued source variable. Thus the source blocks are treated as the two sources in the MAR-PID computation. One could alternatively use the individual source bits as the source universe; then the positive synergistic contribution for target bit $C_{j}$ would be localized at the corresponding pair of source bits and would project back to the original-source atom $\left\{\left\{V_{1},V_{2}\right\}\right\}$.

Let

$$C_{j}=B_{t,j}=b_{t+j}⊕c_{t+j},\;0\leq j\leq k-1.$$

Then

$$I\left(V_{1};C_{j}\right)=0,\;I\left(V_{2};C_{j}\right)=0,\;I\left(\left(V_{1},V_{2}\right);C_{j}\right)=1.$$

For each binary target bit $C_{j}$, the nonzero MAR-PID contribution is the synergistic atom corresponding to the joint source:

$$\Delta I\left(\alpha_{\mathrm{syn}};C_{j}\right)=1,\;\Delta I\left(\alpha ;C_{j}\right)=0\;\mathrm{for}\;\alpha \neq \alpha_{\mathrm{syn}}.$$

Aggregating over the *k* target bits gives

$$\Delta I\left(\alpha_{\mathrm{syn}};Z_{t}^{\left(k\right)}\right)=\sum_{j=0}^{k-1}\Delta I\left(\alpha_{\mathrm{syn}};C_{j}\right)=k.$$

Thus the negative interaction information is replaced by a nonnegative atomic description: one bit of synergistic information is present at each resolved target digit (Table 1).

### 6.2. A Mixed Redundancy–Synergy Example

Let A∼Bern(1/2) be the binary target, and let B∼Bern(q), 0<q<1, be an independent latent switching variable. The observed sources are constructed as follows:

- If B=0, set $V_{1}=A$ and $V_{2}=A$. This regime introduces a redundant mechanism, since either source alone determines the target.
- If B=1, sample U∼Bern(1/2) independently of *A*, and set $V_{1}=U$ and $V_{2}=U⊕A$. This regime introduces an XOR-type synergistic mechanism, since neither source alone determines the target, while the pair does.
- Let $V_{3}$ be either independent noise or a noisy copy of *A*, depending on whether one wants to include an additional weakly informative source.

Since *B* is latent, MAR-PID is applied to the marginal distribution of $\left(A,V_{1},V_{2},V_{3}\right)$, after averaging over the switch. The resulting atoms need not decompose additively into the two latent regimes. The example should therefore be read as a qualitative mechanism test case: one regime introduces redundant information, while the other introduces XOR-type synergistic information. The redundant atom associated with $\left\{\left\{V_{1}\right\},\left\{V_{2}\right\}\right\}$ and the synergistic atom associated with $\left\{\left\{V_{1},V_{2}\right\}\right\}$ are the principal atoms to inspect. Additional atoms involving $V_{3}$ may appear if $V_{3}$ carries information about *A*.

For reference, Table 2 shows population Shannon summaries for the two-source version of this example, without the optional source $V_{3}$. The table is not a MAR-PID benchmark. It only records familiar information quantities for several values of the switch parameter *q*. The change of sign of $I(A;V_{1};V_{2})$ shows how the same family moves from a redundancy-dominated regime to an XOR-type synergy-dominated regime.

### 6.3. Discrete Variable with More than 2 Values

Let $C_{1},C_{2}$ be independent Bernoulli(1/2) variables and let

$$Y=2C_{1}+C_{2}\in \left\{0,1,2,3\right\}.$$

Let $V_{1}=C_{1}$ and $V_{2}=C_{2}$. Then *Y* is a four-valued target, but its quantile binary representation consists of the two independent bits $C_{1}$ and $C_{2}$. MAR-PID applied to $C_{1}$ assigns one bit uniquely to $V_{1}$, while MAR-PID applied to $C_{2}$ assigns one bit uniquely to $V_{2}$. Aggregation over target bits therefore gives two separate original-target information contributions rather than a single undifferentiated two-bit dependence.

The example also shows where representation dependence enters. Internal permutations of bits within a source variable are removed by the projection $\pi^{*}$, so they do not change the resulting original-variable source atom. On the target side, however, the chosen binary representation matters: a substantially different binarization of *Y* may lead to a different target-bit summary. This is why the proposed quantities are finite-resolution summaries induced by a specified target representation.

Together, the examples illustrate the role of the finite-resolution representation. The XOR example starts from continuous variables on [0,1), but MAR-PID is applied only after resolving binary coordinates. The four-valued example shows that target binarization can expose separate informational components that would otherwise appear as a single multivalued dependence.

## 7. Discussion

The present paper separates two tasks that are often conflated: constructing a nonnegative empirical information decomposition, and deriving support- or scale-based summaries from it. We focus on the first task.

The main construction is a finite-resolution empirical MAR-PID pipeline. Continuous or high-cardinality variables are represented by binary coordinates using recursive quantile binarization. MAR-PID is then applied to binary target components, producing nonnegative bit-level atoms. These atoms are aggregated across target bits and lifted from source-bit atoms back to atoms over the original source variables. Thus the binary coordinates serve as computational intermediates rather than final interpretive units.

The finite-depth dyadic property justifies the use of this binary representation. Under non-atomic conditional laws, the binary tree coordinates are balanced and conditionally independent at each finite-tree level. In the present construction, the source side is resolved over the chosen binary source universe: for each target bit $C_{\ell }$, MAR-PID is computed over B, and the resulting source-bit atoms are pushed forward to atoms over the original variables. On the target side, the construction remains MAR-PID-nonnegativity-compatible bit by bit. Each $\Delta I(\alpha ;C_{\ell })$ is a genuine nonnegative binary-target MAR-PID atom, and summing over *ℓ* gives a nonnegative finite-resolution target-bit summary.

The finite-resolution pipeline is neutral with respect to the redundancy–synergy interpretation of the atoms. Once the MAR-PID atoms have been estimated, the same aggregation and lifting procedure applies to atoms interpreted as redundant, unique, or synergistic contributions. Accordingly, the proposed summaries should be interpreted as finite-resolution MAR-PID summaries induced by the chosen binary representation, rather than as a representation-independent continuous-variable PID.

The examples illustrate why the atomic decomposition matters. In the XOR example, classical interaction information is negative, while MAR-PID identifies a nonnegative synergistic contribution at each target bit. In the mixed example, redundant and synergistic mechanisms coexist. A signed net quantity may obscure this coexistence, whereas MAR-PID keeps the mechanisms separated.

The construction has several practical constraints. Finite-sample estimation of high-dimensional conditional channels is statistically demanding. In applied work, one may need restricted atom families, problem-specific source selection, or other regularized channel estimators. Such choices concern large-scale implementation and statistical stabilization; they are not part of the finite-resolution MAR-PID construction itself.

Support-based degrees of freedom, persistent supports, and PID-based dimensions are natural downstream constructions. Once nonnegative empirical atoms are available, one may threshold their support, normalize across source and target resolutions, or study scaling with resolution. These choices require separate treatment. The role of the present paper is to establish the empirical nonnegative MAR-PID layer on which such summaries can be built.

The present paper should be read as a construction paper. It defines a finite-resolution representation, applies MAR-PID at the binary target-component level, and aggregates the resulting nonnegative atoms back to original variables. It does not claim to provide a complete statistical validation procedure. Real-data benchmarking, confidence statements, optimal resolution selection, and large-scale sparse implementation require further statistical and computational choices. These are important for applications, but they are not part of the finite-resolution MAR-PID construction itself.

The full-lattice version is combinatorial. If MAR-PID is computed over *m* source coordinates, there are $2^{m}-1$ nonempty source subsets, and the number of nonempty antichain atoms is M(m)−2, where M(m) is the *m*-th Dedekind number. This gives 4,18,166, and 7579 atoms for m=2,3,4, and 5, respectively. In the bit-level construction, *m* may be $\left|B\right|=\sum_{i}k_{i}$, so source-bit unfolding can increase the lattice quickly. Full-lattice computation should therefore be regarded as a small-source construction. For larger systems, one may reduce the source universe by grouping source bits into original variables, limiting the maximum source order, preselecting variables, or using a problem-specific family of source groups. Pruning very small atoms can be useful as a reporting or post-processing step, but it is not a substitute for the exact Möbius inversion on the chosen lattice.

## Figures and Tables

**Table 1:** MAR-PID structure of the XOR example at resolution $\delta =2^{-k}$.

Atom α	Type	Aggregated Contribution
$\left\{\left\{V_{1},V_{2}\right\}\right\}$	Synergistic	*k*
$\left\{\left\{V_{1}\right\}\right\}$	Unique	0
$\left\{\left\{V_{2}\right\}\right\}$	Unique	0
$\left\{\left\{V_{1}\right\},\left\{V_{2}\right\}\right\}$	Redundant	0

**Table 2:** Population Shannon summaries for the mixed redundancy–synergy example without the optional source $V_{3}$. Information is measured in bits.

*q*	$I(A;V_{1})$	$I(A;V_{2})$	$I(A;V_{1},V_{2})$	$I(A;V_{1};V_{2})$
0	1.000	1.000	1.000	1.000
0.25	0.456	0.456	0.741	0.172
0.50	0.189	0.189	0.656	−0.278
0.75	0.046	0.046	0.697	−0.605
1	0.000	0.000	1.000	−1.000

## Data Availability

No new data were created or analyzed in this study.

## Abbreviations

The following abbreviations are used in this manuscript:

MAR-PID	Mages–Anastasiadi–Rohner partial information decomposition
PID	Partial information decomposition

## Nomenclature

**Symbol**	**Object**
T,Y	Target variables
$C_{\ell }$	Target bit
$X_{i},V_{i}$	Source variables
$B_{i,r}$	Source bit
$K,k_{i}$	Resolution depths
S	Source family
A(V)	Atom lattice
α,γ	Atom indices
$\kappa_{S}$	Source channel
$\Delta I(\alpha ;C_{\ell })$	Bit-level atom
$\pi^{*}\left(\alpha \right)$	Projected atom

## Appendix A. Lattice Formalism for Atoms and the Synergy Order

This appendix summarizes the lattice notation used in the MAR-PID construction.

### Appendix A.1. Sources and Atoms

Let

$$V=\{V_{1},\ldots ,V_{n}\}$$

be the set of observed variables. A source is a nonempty subset of *V*. We denote the set of all sources by

S=P(V)∖{∅}.

An atom is an antichain in S. Thus an atom is a collection α⊆S such that

$$S_{a},S_{b}\in \alpha ,\;S_{a}\neq S_{b}\;⟹\;S_{a}\neg \subset S_{b}\;\mathrm{and}\;S_{b}\neg \subset S_{a}.$$

The set of all atoms is denoted by A(V).

### Appendix A.2. The Synergy/Loss Order

The synergy, or loss, order ${⪯}_{S}$ on A(V) is defined by

$$\alpha {⪯}_{S}\beta \;⟺\;\forall S\in \alpha \;\exists S^{\prime }\in \beta \;\mathrm{such}\;\mathrm{that}\;S\subseteq S^{\prime }.$$

Equipped with this order, A(V) is a finite lattice. The order is used to define cumulative loss and to perform Möbius inversion.

### Appendix A.3. Möbius Inversion

Let F:A(V)→R be a real-valued function. Its Möbius inverse ΔF with respect to ${⪯}_{S}$ is defined recursively by

$$\Delta F\left(\alpha \right)=F\left(\alpha \right)-\sum_{\beta {≺}_{S}\alpha }\Delta F\left(\beta \right),$$

or equivalently

$$F\left(\alpha \right)=\sum_{\beta {⪯}_{S}\alpha }\Delta F\left(\beta \right).$$

In MAR-PID, *F* is the cumulative loss $I_{\cup ,f}\left(\alpha ;T\right)$, and ΔF yields the partial information atoms $\Delta I_{\cup ,f}\left(\alpha ;T\right)$.

## Appendix B. Binary-Input Channels, Blackwell Order, and Zonogons

This appendix recalls the channel-theoretic objects used in the MAR-PID construction. The full proof of nonnegativity is credited to Mages, Anastasiadi, and Rohner [1].

### Appendix B.1. Binary-Input Channels

Assume T∈{0,1}. A binary-input channel κ:T→S is specified by

κ(s∣t)=P(S=s∣T=t).

Equivalently, it can be represented by column vectors

$$v_{s}=\left(\begin{array}{c} \kappa (s|1)\\ \kappa (s|0) \end{array}\right),\;s\in A_{S}.$$

### Appendix B.2. Blackwell Order

For two channels $\kappa_{1}:T\to S_{1}$ and $\kappa_{2}:T\to S_{2}$ with the same input alphabet, write

$$\kappa_{1}{⪯}_{\mathrm{BW}}\kappa_{2}$$

if $\kappa_{1}$ is a garbling of $\kappa_{2}$; i.e., if there exists a stochastic post-processing channel $K:S_{2}\to S_{1}$ such that

$$\kappa_{1}=K\circ \kappa_{2}.$$

Thus $\kappa_{2}$ is at least as informative as $\kappa_{1}$ for all decision problems with input *T*.

### Appendix B.3. Zonogon Representation

For binary-input channels, associate to κ the zonogon

$$Z\left(\kappa \right)=\sum_{s\in A_{S}}\left[0,v_{s}\right]\subset R^{2},$$

a Minkowski sum of line segments. The Blackwell order, joins, and meets can be expressed geometrically in terms of these zonogons. This representation is both computationally useful and central to the proof of the nonnegativity of the MAR-PID atoms.

### Appendix B.4. Nonnegativity Mechanism

For an atom α, define

$$\kappa_{\vee }\left(\alpha \right)=\underset{S\in \alpha }{⋁}\kappa_{S}.$$

The cumulative loss functional is

$$I_{\cup ,f}\left(\alpha ;T\right)=i_{f}\left(p,\kappa_{V}\right)-i_{f}\left(p,\kappa_{\vee }\left(\alpha \right)\right),\;p=P\left(T=1\right).$$

Partial atoms are obtained by Möbius inversion on the synergy/loss lattice. The nonnegativity theorem follows from monotonicity of the channel join, monotonicity of $i_{f}$ under the Blackwell order, and zonogon-based inclusion–exclusion inequality for binary-input channels. Hence, in the binary-target setting,

$$\Delta I_{\cup ,f}\left(\alpha ;T\right)\geq 0\;\forall \alpha \in A\left(V\right).$$

## Appendix C. Finite-Depth Conditional Binarization

In this appendix we prove Proposition 1. We work on standard Borel spaces, so regular conditional distributions exist. Fix n≥0 and write

$$F_{n}=\sigma \left(X_{n}\right).$$

Let

$$\mu_{n}\left(\omega ,\cdot \right)=P\left(X_{n+1}\in \cdot |F_{n}\right)\left(\omega \right)$$

be a regular conditional distribution. We assume that, for almost every ω, the conditional law $\mu_{n}\left(\omega ,\cdot \right)$ is non-atomic on the cells generated below. This guarantees that each cell can be split into two subcells of equal conditional probability. It also avoids ambiguity from atoms or ties. In empirical implementations with ties, one must specify a deterministic or randomized tie-breaking rule; this is a numerical convention and is not part of the population statement proved here.

For each k≥0 and each binary word

$$\beta =\left(b_{1},\ldots ,b_{k}\right)\in {\left\{0,1\right\}}^{k},$$

let $C_{\beta }^{\left(k\right)}\left(\omega \right)$ denote the corresponding depth-*k* cell. These cells are allowed to depend on ω, through the conditional law given $F_{n}$. At depth zero set

$$C_{\emptyset }^{\left(0\right)}=\left[0,1\right].$$

If $C_{\beta }^{\left(k\right)}$ has been defined, choose an $F_{n}$-measurable conditional median $m_{\beta }^{\left(k\right)}$ inside $C_{\beta }^{\left(k\right)}$ such that the two children

$$C_{\beta 0}^{\left(k+1\right)}=C_{\beta }^{\left(k\right)}\cap \left(-\infty ,m_{\beta }^{\left(k\right)}\right],\;C_{\beta 1}^{\left(k+1\right)}=C_{\beta }^{\left(k\right)}\cap \left(m_{\beta }^{\left(k\right)},\infty \right)$$

have equal conditional probability given $F_{n}$ and $X_{n+1}\in C_{\beta }^{\left(k\right)}$. Such measurable choices are standard under the non-atomic conditional-law assumption; alternatively, the argument may be read conditional on a fixed ω outside a null set.

Define the cylinder events

$$E_{\beta }^{\left(k\right)}=\left\{B_{1}^{n+1}=b_{1},\ldots ,B_{k}^{n+1}=b_{k}\right\}=\left\{X_{n+1}\in C_{\beta }^{\left(k\right)}\right\}.$$

**Proof of Proposition 1.** We first show by induction that

$$P\left(E_{\beta }^{\left(K\right)}|F_{n}\right)=2^{-K}$$

for every word $\beta \in {\left\{0,1\right\}}^{K}$. For K=1, this is exactly the first conditional median split. Assume it holds at depth *K*. For $\beta \in {\left\{0,1\right\}}^{K}$ and b∈{0,1},

$$E_{\beta b}^{\left(K+1\right)}\subseteq E_{\beta }^{\left(K\right)},$$

and the defining split yields

$$P\left(E_{\beta b}^{\left(K+1\right)}|F_{n},E_{\beta }^{\left(K\right)}\right)=\frac{1}{2}.$$

Therefore

$$P\left(E_{\beta b}^{\left(K+1\right)}|F_{n}\right)=\frac{1}{2}\;2^{-K}=2^{-(K+1)}.$$

This completes the induction. For each *i* and b∈{0,1}, the event $\left\{B_{i}^{n+1}=b\right\}$ is the disjoint union of $2^{i-1}$ cylinder events of length *i*, each of conditional probability $2^{-i}$. Hence

$$P\left(B_{i}^{n+1}=b|F_{n}\right)=\frac{1}{2}.$$

Finally, for any word $\left(b_{1},\ldots ,b_{K}\right)$,

$$P\left(B_{1}^{n+1}=b_{1},\ldots ,B_{K}^{n+1}=b_{K}|F_{n}\right)=2^{-K}$$

and

$$\prod_{i=1}^{K}P\left(B_{i}^{n+1}=b_{i}|F_{n}\right)=2^{-K}.$$

Thus $B_{1}^{n+1},\ldots ,B_{K}^{n+1}$ are conditionally independent given $F_{n}$. □
